# Anti-Müllerian hormone: a new actor of sexual dimorphism in pituitary gonadotrope activity before puberty

**DOI:** 10.1038/srep23790

**Published:** 2016-03-31

**Authors:** Ghislaine Garrel, Chrystèle Racine, David L’Hôte, Chantal Denoyelle, Céline J. Guigon, Nathalie di Clemente, Joëlle Cohen-Tannoudji

**Affiliations:** 1 Université Paris-Diderot, Sorbonne Paris Cité, Biologie Fonctionnelle et Adaptative (BFA), F-75013 Paris, France; 2 Centre National pour la Recherche Scientifique (CNRS) UMR 8251, Paris, France; 3 Institut National de la Santé et de la Recherche Médicale (INSERM) U1133 Physiologie de l’axe gonadotrope, Paris, France

## Abstract

Anti-Müllerian hormone (AMH) contributes to male sexual differentiation and acts on gonads of both sexes. Identification of AMH receptivity in both pituitary and brain has led to the intriguing idea that AMH participates to the hypothalamic-pituitary control of reproduction, however *in vivo* experimental evidence is still lacking. We show that AMH stimulates secretion and pituitary gene expression of the gonadotropin FSH *in vivo* in rats. AMH action is sex-dependent, being restricted to females and occurring before puberty. Accordingly, we report higher levels of pituitary AMH receptor transcripts in immature females. We show that AMH is functionally coupled to the Smad pathway in LβT2 gonadotrope cells and dose-dependently increases *Fshb* transcript levels. Furthermore, AMH was shown to establish complex interrelations with canonical FSH regulators as it cooperates with activin to induce *Fshb* expression whereas it reduces BMP2 action. We report that GnRH interferes with AMH by decreasing AMH receptivity *in vivo* in females. Moreover, AMH specifically regulates FSH and not LH, indicating that AMH is a factor contributing to the differential regulation of gonadotropins. Overall, our study uncovers a new role for AMH in regulating gonadotrope function and suggests that AMH participates in the postnatal elevation of FSH secretion in females.

Pituitary gonadotropins, Luteinizing hormone (LH) and Follicle-stimulating hormone (FSH), are crucial for mammalian reproductive development and function as they control gonadal steroidogenesis and gametogenesis. Gonadotropins are secreted by gonadotrope cells of the pituitary and are heterodimeric glycoproteins composed of a common α subunit (CGA) dimerized with a specific, rate-limiting β-subunit, LHβ or FSHβ. Pituitary gonadotrope activity is mainly regulated by the hypothalamic Gonadotropin-releasing hormone (GnRH), which stimulates the biosynthesis of the three constituting subunits as well as LH and FSH release^1^,^2^,^3^. Other endocrine or paracrine/autocrine signals contribute to refine the regulation exerted on gonadotropin synthesis^4^,^5^. Among these factors, members of the transforming growth factor-β (TGFβ) superfamily such as activin, inhibin and bone morphogenetic protein-2 (BMP2) and 4 (BMP4), have the ability to increase FSH release and *Fshb* expression, and are thus believed to contribute to the differential regulation of gonadotropins^6^,^7^,^8^.

Anti-Müllerian hormone (AMH), also called Müllerian inhibiting substance, is another member of the TGFβ superfamily. AMH was first identified as a key factor in male sex differentiation^9^,^10^. Secreted by fetal testis, AMH indeed induces the regression of the Müllerian ducts, the anlage of the female reproductive tract. AMH was later involved in the regulation of steroidogenesis in gonads from both sexes^9^,^10^,^11^. The characteristic of AMH secretion is to be highly sexually dimorphic, especially before puberty^9^. In males, high levels of AMH are secreted during embryonic development with levels remaining elevated after birth. AMH then declines at puberty and becomes undetectable in adulthood^12^,^13^. In contrast, circulating levels of AMH originating from ovarian growing follicles in females are reduced as compared to males throughout the immature period^10^,^12^. AMH can still be detected in females during adulthood and serum AMH is now recognized as a reliable marker of ovarian follicular reserve and of ovarian responsiveness to infertility treatment^9^,^10^,^14^,^15^. AMH receptivity was initially thought to be specific of gonads and reproductive organs, leading to the idea that biological effects of AMH would be more restricted than other TGFβ family members. Since the early 2000’s, however, several reports indicated that transcripts encoding the AMH specific type II receptor (AMHR2) are also present into the brain, notably in the hypothalamus and in motor or cortical neurons, as well as in the pituitary gland^16^,^17^,^18^,^19^. AMH was observed to support survival of embryonic motor and cortical neurons^17^,^18^. In the murine immortalized LβT2 gonadotrope cell line, AMH was shown to increase *Fshb* transcripts levels but was inefficient to enhance *Fshb*-promoter-driven luciferase activity^16^. Altogether, this suggests that AMH, in addition to targeting gonads, may participate to the hypothalamic-pituitary control of reproduction. The objective of our study was to characterize AMH effect on pituitary gonadotrope function in male and female rats and to analyze mechanisms of AMH action in LβT2 gonadotrope cells.

## Results

### AMH activates the Smad signaling pathway exclusively in the pituitary gonadotrope cell lineage

**Figure 1:**
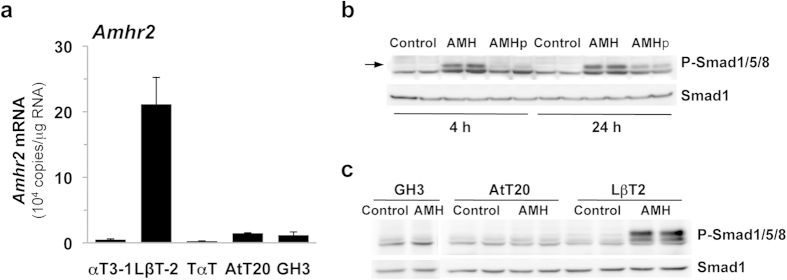
*Amhr2* is highly expressed in gonadotrope cell lineage and AMH recruits the Smad signaling pathway in LβT2 cells. Quantification of *Amhr2* transcripts in representative cell lines of the different anterior pituitary cell types. Levels of *Amhr2* mRNA were measured by Taqman real-time qPCR in the gonadotrope αT3-1 and LβT2, thyrotrope TαT-1, corticotrope AtT20 and somatolactotrope GH3 cell lines. Data are the mean ± SEM of 3 independent cultures. (**b**) Time course of AMH precursor (AMHp) and cleaved AMH (AMH) effects on Smad1/5/8 phosphorylation in the gonadotrope LβT2 cell line. Cells were treated for 4 or 24 h with 2.5 μg/ml (17.5 nM) AMHp or AMH (concentrations conventionally used in AMH *in vitro* studies) and Phospho-Smad1/5/8 (P-Smad1/5/8) protein level was evaluated by immunoblotting. Total Smad1 was used as a loading control. (**c**) No effect of AMH on Smad1/5/8 phosphorylation in the corticotrope AtT20 and the somatotrope GH3 cell lines. Cells were treated for 4 h with 2.5 μg/ml AMH and Phospho-Smad1/5/8 (P-Smad1/5/8) protein level was evaluated as described above. The LβT2 cell line is used as a positive control.

To evaluate the pituitary lineages expressing *Amhr2*, real-time quantitative PCR (qPCR) analysis was performed on representative cell lines of the different pituitary cells types (Fig. 1a). *Amhr2* transcripts could be detected in the murine immortalized LβT2 gonadotrope cells but not in the less differentiated αT3-1 gonadotrope cells, as previously reported^16^. Absolute quantification of *Amhr2* mRNA revealed an average number of 2.1 · 10^5^ copies/μg RNA in LβT2 cells. No *Amhr2* transcript could be amplified in TαT-1 thyrotrope cells. Its expression level in AtT20 corticotrope and GH3 somatotrope cell lines was about 20 fold lower than in LβT2 cells. We next examined the activation by AMH of its canonical Smad1/5/8 pathway^9^. AMH is synthetized as a homodimeric glycoprotein (AMH precursor, 140 kDa). AMH is cleaved to yield a 25-kDa C-terminal dimer and a 110-kDa N-terminal dimer, which remain associated as a noncovalent complex (cleaved AMH). Both the cleaved noncovalent complex (140 kDa) and the C-terminal dimer are receptor binding and active forms^20^. Cells were stimulated for 4 or 24 h either with 2.5 μg/ml (17.5 nM) of AMH precursor or cleaved noncovalent complex of AMH, a concentration conventionally used in *in vitro* studies^11^,^16^. Immunoblotting with antibodies recognizing the phosphorylated forms of Smad1/5/8 revealed (Fig. 1b) that P-Smad1/5/8 could be barely detected in LβT2 cells in basal conditions. AMH significantly enhanced the levels of P-Smad1/5/8 at 4 and 24 h, demonstrating that AMH is functionally coupled to the Smad pathway in gonadotrope cells. Both AMH forms were able to stimulate the Smad pathway although AMH precursor was less efficient than the cleaved form, especially at 4 h. Although small levels of *Amhr2* transcript were detected in AtT20 and GH3 cells, the Smad1/5/8 pathway was not activated by AMH in these cell lines (Fig. 1a,c) indicating that functional AMH receptivity is restricted to the gonadotrope lineage.

### AMH increases *Fshb* expression in LβT2 gonadotrope cells

**Figure 2:**
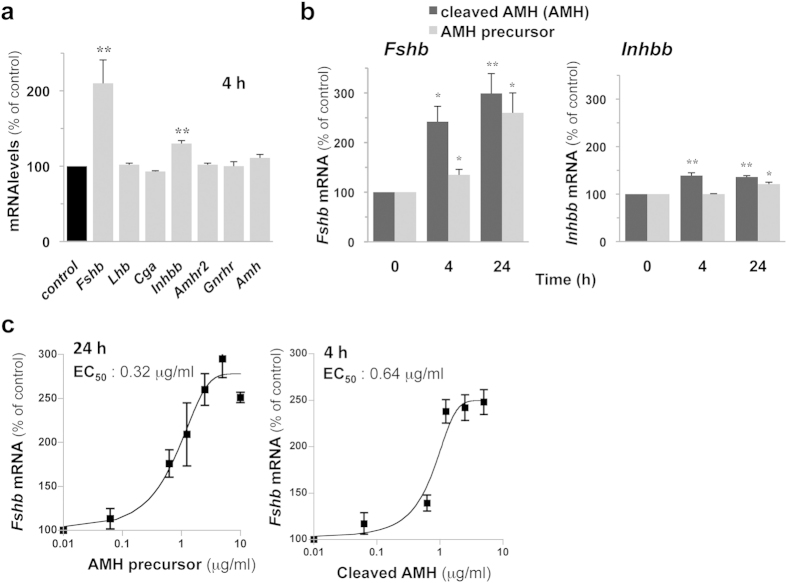
*Fshb* and *Inhbb* are two target genes of AMH in gonadotrope LβT2 cells. (**a**) AMH selectively stimulates *Fshb* and *Inhbb* expression in LβT2 cells. Cells were stimulated with 2.5 μg/ml AMH for 4 h and transcripts levels were determined by real time qPCR. (**b**) Time course of AMH precursor and AMH effects on *Fshb* and *inhbb* transcripts levels. LβT2 cells were treated for 4 or 24 h with 2.5 μg/ml AMH precursor or AMH. (**c**) Dose-response of AMH on *Fshb* transcript levels. LβT2 cells were treated for 4 h with increasing concentrations (0.06 to 10 μg/ml) of AMH precursor or AMH. *Fshb* mRNA levels were expressed as percentage of control. Data are the mean ± SEM of 3 to 10 experiments. *P ≤ 0.05; **P ≤ 0.001 compared with untreated cells (control).

In order to identify AMH target genes in gonadotrope cells, LβT2 cells were stimulated for 4 h with AMH and levels of several transcripts were analyzed by real-time quantitative PCR (qPCR). Among these genes, transcript levels of *Fshb* and, to a lesser extent, *Inhbb* encoding the β-subunit of activin and inhibin, were significantly increased by AMH treatment (Fig. 2a). Levels of *Lhb* and *Cga* transcripts as well as transcripts of *Amhr2* and *Gnrhr* were unaffected at 4 h (Fig. 2a). Transcript levels remained unaffected after a 24 h treatment (104 ± 7, 104 ± 4 and 93 ± 5% of control, for *Lhb, Amhr2* and *Gnrhr,* respectively. data not shown). No significant change in *Amh* expression was detected indicating that no autoregulatory loop of *Amh* expression is operative in these cells (Fig. 2a). Both forms of AMH significantly enhanced *Fshb* and *Inhbb* transcript levels and their effects were similar after 24 h (Fig. 2b). AMH precursor, however, displayed a less potent effect after a short 4 h-period of stimulation. Stimulation of cells with increasing concentrations of AMH precursor significantly increased *Fshb* expression in a dose-dependent manner (Fig. 2c). A similar profile was obtained when stimulating LβT2 cells with AMH precursor for 24 h or with cleaved AMH for 4 h, suggesting that gonadotrope cells can process AMH precursor during the 24 h incubation to generate a cleaved active form. No effect could be observed on *Lhb* transcript levels, whatever the time or the form of AMH used (data not shown).

### AMH synergistically cooperates with activin but not with BMP2 to increases *Fshb* expression in LβT2 cells

**Figure 3:**
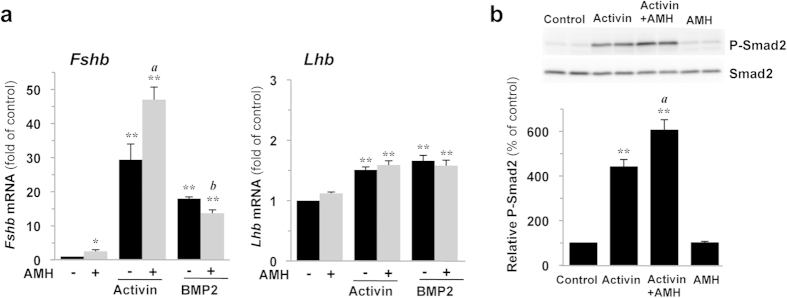
AMH potentiates activin signaling and activin effect on *Fshb* expression in LβT2 cells. LβT2 cells were stimulated for 4 h with 2.5 μg/ml AMH alone or combined with 10 ng/ml activin A or 20 ng/ml BMP2. (**a**) AMH enhances activin and counteracts BMP2 stimulation of *Fshb* expression. *Fshb* and *Lhb* mRNA levels were determined by real-time qPCR and expressed as the mean ± SEM of at least 3 independent experiments. (**b**) AMH improves activin coupling to the Smad2/3 pathway. P-Smad2 protein level was evaluated by immunoblotting and normalized with total Smad2. *P ≤ 0.05; **P ≤ 0.01 compared with control cells. *a*, P ≤ 0.05 between AMH and AMH+activin treatments. *b*, P ≤ 0.05 between AMH and AMH+BMP2 treatments.

We next determined whether AMH could modulate the action of other known potent inducers of *Fshb* expression such as activin or BMP2. Activin and BMP2 have been reported to activate the Smad2/3 and the Smad1/5/8 respectively in LβT2 cells^7^,^21^. As expected, a 4 h-treatment of LβT2 cells with activin or BMP2 significantly increased *Fshb* transcript levels (by 29 ± 5 and 18 ± 1 fold, respectively, Fig. 3a). When cells were co-stimulated with AMH and activin, the combined observed effect was greater than the sum of their individual effects, indicating that AMH synergistically cooperates with activin to induce *Fshb* expression in LβT2 gonadotrope cells. Activin also increased *Lhb* transcripts levels although to a lesser extent than *Fshb* but no synergism could be detected for this gene. The synergism between AMH and activin appears thus to be specific of *Fshb* expression. Contrasting with activin, AMH did not cooperate with BMP2 in inducing *Fshb* as no synergistic or additive effects could be detected. Instead, AMH inhibited by 25% the action of BMP2 (Fig. 3a). To better understand the mechanisms underlying AMH and activin synergism, we next measured activin signaling in cells co-treated or not with AMH. Although AMH alone did not affect Smad2 phosphorylation, it significantly increased activin-induced P-Smad2 levels (Fig. 3b). Same results were obtained after detection and quantification of P-Smad3 levels (activin-induced phosphorylation of Smad3 was increased by 1.4 ± 0.1 fold in presence of AMH, data not shown). In contrast, no effect of AMH on BMP2 induced phosphorylation of Smad1/5/8 could be detected (1.08 ± 0.03 fold over BMP2, data not shown). Altogether, this indicates that AMH, in addition to its proper effects, potentiates activin signaling and activin-dependent *Fshb* expression.

### GnRH counteracts AMH action by decreasing AMH receptivity both *in vitro* and *in vivo*

**Figure 4:**
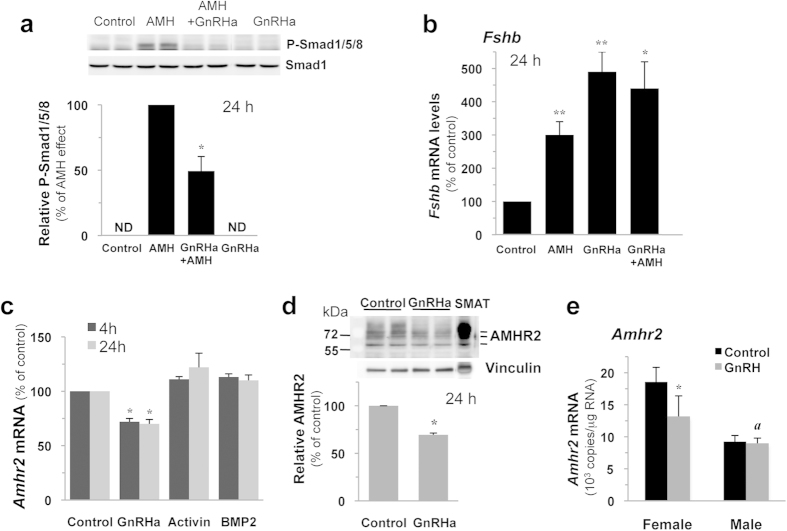
GnRH down-regulates AMH receptivity *in vitro* in LβT2 cells and *in vivo* in pituitary of female rats at pnd 18. (**a**) GnRH inhibits AMH signaling. LβT2 cells were stimulated for 24 h with 2.5 μg/ml AMH, 10 nM GnRHa or with a combination of both hormones. P-Smad1/5/8 protein level was evaluated by immunoblotting and normalized with total Smad1. *P ≤ 0.05 compared with AMH-stimulated cells. (**b**) Combined effects of GnRH and AMH on *Fshb* expression. Cells were incubated with 2.5 μg/ml AMH, 10 nM GnRHa or with both hormones for 24 h. *Fshb* mRNA levels were analyzed by real-time qPCR and expressed as percentage of control levels. *P ≤ 0.05 ; **P ≤ 0.01 compared with control cells. (**c**) GnRH down-regulates *Amhr2* expression. Cells were incubated with 10 nM GnRHa, 10 ng/ml activin A or 20 ng/ml BMP2 for 4 and 24 h. *Amhr2* mRNA levels were determined by real-time qPCR and expressed as percentage of control cells. Results are the mean ± SEM of 8 independent experiments. *P ≤ 0.05 compared with control cells. (**d**) GnRH decreases AMHR2 protein level. Cells were treated for 24 h with 10 nM GnRHa and AMHR2 protein level was determined by immunoblotting after normalization with vinculin. Results are the mean ± SEM of 4 independent experiments. *P ≤ 0.05 compared with control cells. (**e**) GnRH down-regulates *Amhr2* expression in pituitary of female rats at pnd 18. Male and female rats were injected subcutaneously at pnd 17 with 100 μl of saline solution containing or not 0.1 μg of GnRHa. Anterior pituitary *Amhr2* expression was determined 24 h after injection by Taqman real time qPCR. Each value is a mean ± SEM of 8 to 14 rats. *P ≤ 0.05 compared to control rats; *a*, P ≤ 0.01 compared to female control rats.

GnRH being the main regulator of gonadotrope function, we next analyzed whether GnRH could interfere with the action of AMH. Immunoblotting analyses showed that the activation of Smad 1/5/8 pathway by AMH was markedly blocked (51 ± 11% inhibition) in the presence of the GnRH agonist (GnRHa) Triptorelin in LβT2 gonadotrope cells (Fig. 4a). The consequence of such decrease was studied on the regulation of *Fshb* expression by AMH. No additional increase in *Fshb* expression could be detected when AMH was added to GnRHa although AMH alone significantly enhanced *Fshb* transcript levels (Fig. 4b). We next measured the expression of *Amhr2* transcripts and protein after GnRHa treatment. The number of *Amhr2* transcript copies was not significantly affected when cells were treated either by activin or BMP2 for 4 or 24 h (Fig. 4c). In contrast, treatment with GnRHa significantly reduced this level at 4 h and this inhibition was maintained at 24 h. Although modest, the observed inhibitory effects (28 ± 3% at 4 h and 30 ± 4% at 24 h) were highly reproducible (Fig. 4c). Immunoblotting using a monoclonal antibody directed against AMHR2 extracellular domain revealed three bands of approximately 63, 71 and 80 kDa that were highly expressed in extracts from the Sertoli cell line SMAT-1 (Fig. 4d). These three molecular forms of AMHR2 were previously identified using a HA-tagged AMHR2^22^. Quantification of the 63-kDa form revealed a 30 ± 2% decrease in AMHR2 expression following a 24 h GnRHa treatment and inhibition was also observed for the other forms (Fig. 4d). Altogether, these data indicate that GnRH is able to decrease AMH receptivity and thus AMH signaling in LβT2 cells.

To determine whether such regulation could also be observed *in vivo*, pituitary *Amhr2* transcript levels were quantified 24 h after administration of the GnRHa Decapeptyl to immature postnatal day 18 (pnd 18) rats (Fig. 4e). Pituitary *Amhr2* levels were reduced by 30% in females, corroborating the inhibitory effect of GnRH observed *in vitro*. In contrast, pituitary *Amhr2* expression was unaffected in males indicating that such regulation is sexually dimorphic. Noteworthy, *Amhr2* transcript levels were about 2 fold higher in females than in males.

### Sexual dimorphism in pituitary *Amhr2* expression in immature rats

**Figure 5:**
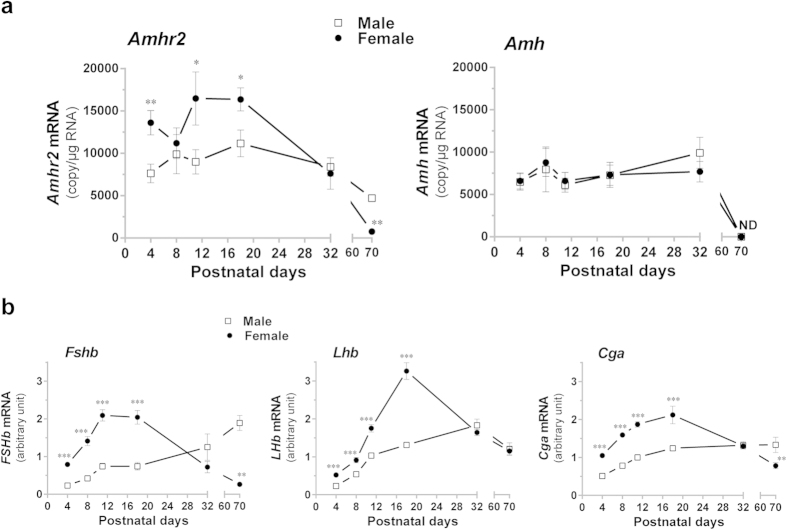
Postnatal ontogeny of *Amh* and *Amhr2* expression in anterior pituitary of male and female rats. Anterior pituitary of male and female Wistar rats were collected at pnd 4, 8, 11, 18, 32 or 70. (**a**) Ontogeny of *Amh* and *Amhr2* expression in rat pituitary. Transcript levels were measured by Taqman real-time qPCR and expressed as the number of *Amh* or *Amhr2* mRNA copies/μg total RNA. ND: non detectable. (**b**) Concomitant pituitary expression of gonadotropin subunit transcripts. Levels of *Fshb, Lhb* and *Cga* mRNA (arbitrary units) were measured throughout the same developmental period. Each value is a mean ± SEM of 6 to 13 rats. *P ≤ 0.05; **P ≤ 0.01 compared to age-matched male rats.

To examine the physiological relevance of the observed *in vitro* effects of AMH, pituitary *Amhr2* mRNA levels were analyzed in adults and in maturing female and male rats at different postnatal periods: neonatal period (first week), infantile period which extends from pnd 7 to pnd 21, and also at pnd 32 corresponding approximately to the end of the juvenile period^23^. The amount of pituitary *Amhr2* mRNA copies was found to be 2-fold higher in females than in males at pnd 4 (7625 ± 1097 vs 13610 ± 1429 copies/μg RNA; Fig. 5a). Thereafter, and until pnd 20, the amount of *Amhr2* mRNA remained higher in females, except at pnd 8. In juvenile rats *i.e.* at pnd 32, sexual dimorphism could no longer be detected. In adults (pnd 70), *Amhr2* mRNA amount was significantly reduced in females and was lower than in male pituitaries. Interestingly, in females, the developmental profile of pituitary *Amhr2* mRNA showed a similar trend as that of gonadotropin subunits mRNA and especially *Fshb* (Fig. 5b). Using the same approach, we quantified *Amh* mRNA in the pituitary of immature females and male rats (Fig. 5a). Although pituitary *Amh* expression was weaker than that of *Amhr2*, this indicates that AMH may act as an autocrine/paracrine regulator in this organ. The number of *Amh* mRNA copies was similar in male and female pituitaries and no significant change was detected throughout the immature period (average number of 7500 copies/μg RNA). Levels became then undetectable in both sexes in adulthood. Serum AMH assays showed that AMH levels were higher in males at pnd 18 (15 ± 1.5 and 0.7 ± 0.2 ng/ml, in males and females, respectively, data not shown). Circulating AMH levels then decreased to reach undetectable values in adult males whereas AMH concentration remained similar in adult females.

### AMH induces *Fshb* expression and FSH secretion in immature females but not male rats

**Figure 6:**
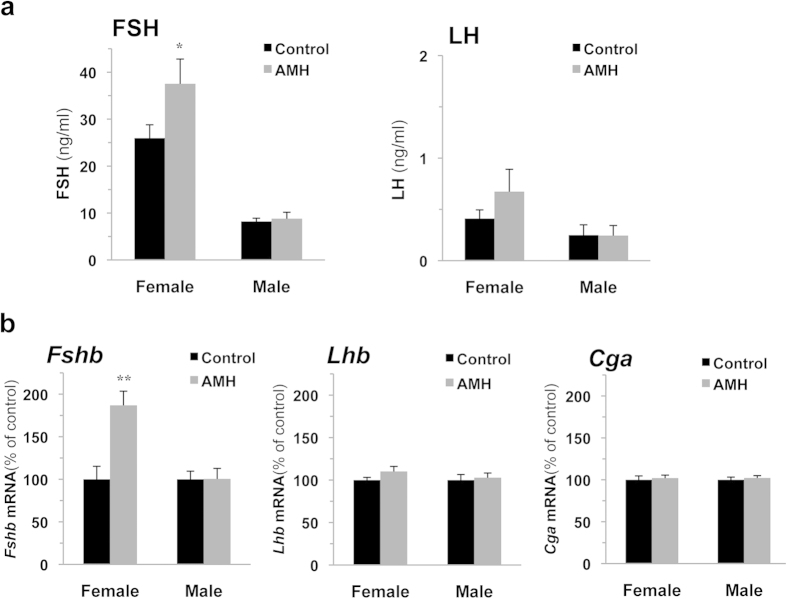
AMH stimulates FSH secretion and pituitary *Fshb* expression, *in vivo*, in female but not in male rats at postnatal day 18. Male and female rats were injected intraperitoneally at pnd 17 with 100 μl of saline solution containing or not 10 μg of AMH precursor (a conventionally used concentration in AMH *in vivo* studies). Serum LH and FSH secretion (**a**) and gonadotropin subunits gene expression (**b**), were determined 18 h after injection. Each value is a mean ± SEM of 13 rats. *P ≤ 0.05; **P ≤ 0.01 compared to control rats.

We next wanted to determine whether AMH could regulate gonadotrope cell activity *in vivo*. A conventionally used dose of 10 μg of AMH precursor^24^,^25^ was intraperitoneally (i.p.) injected to pnd 18 rats of both sexes and serum gonadotropins level was measured 18 h later. AMH treatment significantly increased FSH secretion in females (Fig. 6a), identifying AMH as a new regulator of gonadotrope function. Remarkably, AMH effect was restricted to females as no change in FSH secretion was observed in males. Sexual dimorphic expression of pituitary *Amhr2* would thus be associated with a differential AMH receptivity between immature male and female rats. Noteworthy, AMH did not affect LH secretion either in males or females (Fig. 6a). Further supporting a role of AMH in the regulation of gonadotrope function, we demonstrated that *in vivo* AMH treatment increased female pituitary *Fshb* mRNA (Fig. 6b). Again, this response was sex-dimorphic and restricted to *Fshb* since AMH did not regulate the expression of *Lhb* or *Cga* either in males or in females. Determination of serum steroid levels by gas chromatographic-mass spectrometric analysis did not reveal any significant change in estradiol or testosterone secretion 18 h after AMH administration (estradiol in females: 10 ± 2.8 and 13 ± 2.5 pg/ml; testosterone in males: 0.34 ± 0.13 and 0.23 ± 0.12 ng/ml, before and after AMH treatment respectively, data not shown). AMH thus likely acts directly on pituitary to regulate FSH secretion specifically in females before puberty.

## Discussion

Although AMH receptivity has been identified in both brain and pituitary^16^,^17^,^18^, the ability of AMH to interfere with the hypothalamic-pituitary control of reproduction remained to be determined. By combining *in vitro* and *in vivo* studies in rats, we uncover an original role of AMH in regulating FSH expression and secretion.

The *in vivo* action of AMH on pituitary gonadotrope cells was investigated during the immature period characterized by a production of AMH by gonads of both sexes. Furthermore, during this period, a transient activation of the hypothalamic-pituitary-gonadal axis is critical for the maturation of the brain and other tissues in most mammals. Although being of functional significance, the underlying mechanisms of such activation are still not fully understood^26^. We report that *in vivo* administration of AMH to immature rats increases FSH secretion and expression of pituitary *Fshb* in females 18 h later. Because such treatment did not concomitantly affect ovarian estradiol secretion, observed changes are most likely mediated by a direct effect of AMH on pituitary. Alterations in pituitary gonadotrope activity have already been described in *Amh* knockout mice with decreased serum FSH levels reported in adult females^27^. However, because in these animals disruption of AMH signaling is associated with severe alterations in gonadal activity, it is not possible to assess a direct AMH effect on gonadotrope cells. Further supporting a direct regulation, we provide the first demonstration to our knowledge that AMH couples to its canonical Smad pathway in the LβT2 gonadotrope cell line and we document the AMH induction of *Fshb* expression. The inactive precursor form of AMH is the predominant form secreted by somatic gonadal cells and found into the blood^28^. We show here that LβT2 gonadotrope cells are able to process AMH, consistently with the reported broad expression of subtilisin/kexin-like proprotein convertases family of enzymes, which are responsible for AMH cleavage^29^. Furthermore, because we demonstrate that *Amh* is co-expressed with *Amhr2* in both rat pituitary gland and LβT2 cells, we believe that locally produced AMH may also participate to the regulation of pituitary gonadotrope activity as described for pituitary activin and BMPs^30^. We show here that AMHR2 is functionally coupled to the Smad pathway in LβT2 cells but not in any other pituitary cell lineages indicating that AMH mainly targets gonadotrope cells.

One characteristic of LH and FSH secretion is to be differentially regulated in several physiological conditions^31^. Among the proposed mechanisms is the dynamic change in GnRH pulse frequency with increasing frequencies favoring LH release whereas decreasing frequencies result in a greater FSH release^32^,^33^,^34^,^35^. Another mechanism is the action of activin, which together with inhibin and follistatin, preferentially regulates FSH^4^,^5^,^30^,^36^. We report here that *in vivo* administration of AMH to immature female rats stimulates FSH without affecting LH secretion. Supporting this observation, AMH was found to increase *Fshb* but not *Lhb* transcript levels both *in vivo* and *in vitro*. No change in *Lhb* expression could be detected in LβT2 cells whatever the concentration of AMH used or the duration of stimulation. AMH displays an even higher selectivity toward *Fshb* expression than activin or BMP2 because the latters also increased, although to a lesser extent, *Lhb* mRNA levels, in agreement with previous reports^37^. Furthermore, *Inhbb* was identified as a new AMH target among the few genes known to be regulated by AMH. By increasing *Inhbb* expression and thus possibly pituitary activin B, AMH may reinforce its positive regulation on pituitary FSH expression. Altogether, our study, by pointing out that AMH affects pituitary gonadotrope cells activity, opens a broader field of action of AMH into the organism. Moreover, it reveals AMH as a new factor contributing to the differential regulation of gonadotropins.

Noteworthy, we show that in addition to its own effects on *Fshb*, AMH interacts with other members of the TGFβ family to regulate *Fshb* in LβT2 cells. AMH indeed synergistically cooperates with activin to induce *Fshb* expression and this can be explained, at least in part, by its ability to enhance activin coupling to the Smad2/3 signaling pathway. AMH signaling may improve interaction of Smad2 and Smad3 with activated activin receptors notably through stabilization of Smad adaptators or regulation of the nucleocytoplasmic shuttling of Smads, as reported for TGFβ^38^. That AMH counteracts the action of BMP2 on *Fshb* expression is rather intriguing given that both activate *Fshb* expression. AMH probably affects downstream BMP2 signaling steps because it did not disrupt BMP2 coupling to the Smad pathway. Noteworthy, we show here that GnRH down-regulates endogenously expressed AMHR2 at both the gene and protein levels in LβT2 cells. The mechanisms regulating *Amhr2* expression are still poorly elucidated and only few regulatory hormones have been identified so far. Among them are gonadotropins and estrogens, being reported to reduce *Amhr2* expression in mature follicles in the ovary^39^. An inhibitory effect of LH on *Amhr2* mRNA levels has recently been reported in human granulosa cells^40^. Our cell-based data, thus, adds GnRH to the short list of known regulators of AMHR2 expression and the demonstration *in vivo* of an inhibitory GnRH effect supports the physiological relevance of this observation. GnRH directly regulates gonadotropins biosynthesis and release but also controls numerous signaling and transcriptional factors to orchestrate gonadotrope cells regulation^1^,^35^. That GnRH targets *Amhr2* expression thus further argues for the importance of AMH in regulating gonadotrope function. Overall, our data depict a complex network of interrelations between AMH, locally produced factors and also GnRH that likely contributes to the fine-tuned regulation of pituitary FSH.

We report here that, strikingly, AMH administration increases FSH secretion only in immature females, as serum FSH levels and pituitary *Fshb* expression are unaffected in age-matched males. In line with this observation, we show a sexually dimorphic expression of pituitary *Amhr2* transcripts that closely parallels *Fshb* mRNA expression throughout most of the immature period. We believe that AMH availability is probably not critical for the sex-dependent regulation of FSH. Indeed, during this period, circulating AMH concentrations in rats are lower in females than in males, in agreement with what has been reported in other species^12^,^41^. Moreover, the amount of pituitary *Amh* transcripts was identical in both sexes. Lastly, even with the high dose of AMH administered, FSH biosynthesis and release could not be induced in males. Pituitary AMH receptivity may thus be the limiting factor in the action of AMH on FSH. In several mammalian species, a clear sexually dimorphic feature of the pituitary during the infantile life is the higher serum concentration of FSH in females than in males^23^. The surge in circulating FSH levels, occurring around 15 days of age in female rats, has been associated with a transient elevation of estradiol levels implicated in sexual differentiation of neural circuits generating preovulatory surges as well as in female sexual behavior^42^,^43^. Overall, our data indicates that AMH likely participates in the elevated FSH secretion in prepubertal females. Other regulations from locally produced factors or from GnRH itself, despite its assumed low release before puberty, may also be involved. Studies from Schally’s laboratory^42^,^43^ indeed showed that administration of GnRH to immature females at pnd 15 increased FSH secretion while being ineffective in males. Similarly, a study in humans reported a higher FSH secretion in prepubertal girls than in boys following GnRHa challenge^44^. Consistent with the observed sex-dependent action of AMH at the pituitary level, several studies revealed AMH as a putative factor contributing to brain sexual dimorphism. Disruption of AMH in male mice was indeed associated with subtle feminization of exploratory behaviors^45^ and with alterations in the sexually dimorphic nucleus of the medial preoptic area^46^. We believe that in adulthood, AMH becomes less critical in regulating pituitary gonadotrope activity. Indeed, although AMH is still secreted in adult female rats, *Amhr2* is weakly expressed in the pituitary, suggesting that gonadotrope cells loose AMH receptivity. We speculate from our data that the marked increase of GnRH tonic secretion at puberty is one explanation for such decreased pituitary AMH receptivity. Altogether, our study indicates that AMH, by contributing to transient increments of FSH secretion in immature females, may be critical for proper puberty onset and sexual differentiation. Targeted inactivation of *Amhr2* in pituitary gonadotrope cells should provide a better understanding of the role of pituitary AMH signaling in the regulation of gonadotrope axis maturation and puberty onset in female.

First considered as a key factor of male sexual differentiation, AMH was then identified as a regulator of gonadal function. Our study further extends the role of AMH by showing that it regulates pituitary gonadotrope activity and raises new perspectives in the understanding of central control of reproduction. Because recent studies report that AMH is synthetized by neurons^17^,^18^, it is indeed tempting to speculate that AMH also acts centrally to control GnRH neurons activity. Moreover, the finding that AMH only regulates FSH and that such regulation is sexually dimorphic in immature rats reveals AMH as a key actor in the complex hormonal network preceding puberty in females. Important challenges in the near future are to determine whether alterations of the pituitary AMH system may be related to some puberty disorders.

## Methods

### Materials

AMH precursor (140 kDa) and cleaved AMH (cleaved noncovalent complex of AMH, 140 kDa) were produced as described in Pepinsky *et al*.^47^. The GnRH agonist (GnRHa) Triptorelin and anti-vinculin antibody were from Sigma (V9131; Saint-Quentin Fallavier, France). The GnRHa Decapeptyl was from IPSEN (Boulogne-Billancourt, France). Activin A, bone morphogenetic protein-2 (BMP2) and antibody directed against AMHR2 were from R&D Systems Europe (AF1618, Lille, France). Antibodies against phospho- and total Smads were from Cell Signaling (P-Smad2 #3101, P-Smad1/5/8 #9511, Smad2 #5339; Ozyme, France) except for total Smad1 (ab131371, Abcam, Paris, France).

### *In vivo* experiments

Experiments were conducted according to a protocol that was approved by the institutional animal care and use committee of Paris Diderot University (CEEA40).

Ontogenetic studies: Pregnant Wistar rats were purchased from Janvier Labs (France) and maintained at 24 ± 3 C with a 12 h light/12 h dark cycle. Pups were killed on pnd 4, 8, 11, 18 or 32 (birth designated as pnd 0). Three independent groups of three pregnant females were used. A total of 6 to 13 pups from 6 to 9 different litters were collected for each age point. Adult male and female Wistar rats of 70 days (6 animals per sexe) were from Janvier Labs. Anterior pituitary glands were dissected, deep-frozen in liquid nitrogen and stored at −80 C until RNA extraction.

AMH and GnRH treatments: Male and female rats were injected i.p. at pnd day 17 with 100 μl of saline solution containing or not 10 μg of AMH precursor (the half-life of injected AMH is expected to be of approximately 4 h^48^) or injected subcutaneously with 0.1 μg of the GnRHa, Decapeptyl. Animals were sacrificed after 18 h (AMH) or 24h (GnRHa). Anterior pituitaries were dissected and deep-frozen in liquid nitrogen and sera were stored at −20 C. Six different litters of rats coming from two independent groups of pregnant rats were used for AMH treatment and four different litters of rats for GnRH treatment.

### Cell cultures

Representative cell lines of the different anterior pituitary cell types were used^49^. The thyrotrope TαT-1 and gonadotrope αT3-1 and LβT2 cell lines were generated and kindly provided by Dr P. Mellon. The corticotrope AtT20 cell line were from ATCC and somatolactotrope GH3 (GH3B6 subclone) provided by Danielle Gourdji^50^.

LβT2 cells, generated by Pamela Mellon^51^, were grown to 80% confluence in 12-well plates (1 × 10^6^ per well) as previously described^52^. Cells plated in duplicate were starved overnight in serum-free medium and incubated for 4 or 24 h with 0.06 to 10 μg/ml cleaved AMH (AMH) or AMH precursor. Cells were also stimulated with 2.5 μg/ml (17.5 nM) AMH, a concentration conventionally used in *in vitro* studies^11^,^16^. AMH was given alone or in combination with activin A (10 ng/ml), BMP2 (20 ng/ml) or GnRHa (10 nM).

### Real-time quantitative PCR

Total RNA from cell lines or rat anterior pituitary were isolated with RNeasy-kit (Qiagen) and first-strand cDNA was obtained from 1–2 μg with Superscript II reverse transcriptase (Invitrogen) using random primers according to the manufacturer’s instructions.

Quantification of *Amh* and *Amhr2* mRNA in rat anterior pituitary and LβT2 cells was performed by TaqMan real-time qPCR. *Hprt* was used to normalize RNA expression levels. Primer and UPL probe (Roche Diagnostics) sequences are indicated in Table 1. Real-time qPCR was performed in duplicates with one-fifth dilution of cDNA using the Lightcycler 480 Probes Master kit and were carried out in the LightCycler 480 Instrument (Roche Diagnostics). The qPCR protocol used an initial denaturating step at 95 C for 10 min followed by 45 cycles at 95 C for 10 sec, 60 C for 30 sec and 72 C for 1 sec. Negative controls were run for every primer or probe combination. To generate standard curves, different concentrations of purified and quantified PCR products were amplified.

Quantification of *Lhb*, *Fshb*, *Cga, Inhbb and Gnrhr* mRNA was performed by real-time qPCR using Takyon No ROX SYBR master mix (Eurogentec). Real-time qPCR was carried out in duplicates in LightCycler 480 using 5 μl of the mix containing 0.5 mM of each primer and 5 μl of a 1:20 cDNA dilution. Cycling conditions included an initial heat-denaturing step at 95 C for 3 min, followed by 40 cycles at 95 C for 10 sec, 60 C for 10 sec and 72 C for 10 sec. Oligonucleotide primer sequences are indicated in Table 1 and *Cyclophilin* was used to normalize RNA expression levels. Primer pairs were designed to target cDNA fragments encompassing at least one intron in the gene sequence to prevent amplification of genomic DNA. The specificity of amplification was checked by gel electrophoresis and melting curve analysis. Data were analyzed using the advanced-E-method with standard-curve derived efficiencies obtained from LightCycler 480 software.

### Gonadotropin, AMH and steroid assays

Serum LH and FSH concentrations were determined using rat pituitary magnetic bead panel Milliplex Map kit (RPTMAG-86K-Milliplex). Serum AMH concentrations was determined by ELISA (AMHGenII ELISA, ref A79765, Beckman Coulter). Determination of steroid serum levels was performed by gas chromatographic-mass spectrometric analysis as described in^53^.

### Protein extraction and immunoblotting

Anterior pituitaries and LβT2 cells were homogenized in a buffer containing 10 mM Tris-HCl (pH 7.4), 30 mM NaPPi, 50 mM NaCl, 1% Triton-X100, and 1 mM dithiothreitol supplemented with protease and phosphatase inhibitor cocktails. Homogenates were centrifuged at 20 000 × g for 30 min at 4 C. Equal amounts of protein were separated on a 10% SDS-PAGE. After transfer onto a nitrocellulose membrane, specific antibodies and Pierce ECL2 substrate were used to detect PhosphoSmad2 (P-Smad2), phospho-Smad3 (P-Smad3), P-Smad1/5/8 (final dilution of antibodies, 1:1000). AMHR2 antibody was used at a final concentration of 0.1 μg/ml. Blots were analyzed with a Fuji LAS-4000 imager and quantified using MultiGauje software. Total Smad and vinculin (dilution of antibodies, 1:1000 and 1:30 000, respectively) were used as internal loading control for P-Smad and AMHR2 expression, respectively. Between each detection, membranes were stripped in stripping buffer (2% SDS, 62.5 mM Tris pH 6.8, 114 mM βmercaptoethanol) for 30 min at 50 C.

### Statistical analysis

Statistical differences between age-matched males and females were determined by the non-parametric Mann-Withney test and age differences within the same sex by one-way ANOVA followed by non-parametric Kruskal-Wallis test. For *in vitro* studies in LβT2 cells, experiments were performed in duplicate and all given values are the mean ± SEM of at least 3 independent experiments. Results were analyzed by the non-parametric Mann-Withney test. *P ≤ 0.05 was considered significant.

## Additional Information

**How to cite this article**: Garrel, G. *et al.* Anti-Müllerian hormone: a new actor of sexual dimorphism in pituitary gonadotrope activity before puberty. *Sci. Rep.* **6**, 23790; doi: 10.1038/srep23790 (2016).

## Figures and Tables

**Table 1:** Sequences of the primers and probes used for quantification of transcript levels in rat anterior pituitary, LβT2 cells and other pituitary clonal cell lines.

Target cDNA	Forward primer written 5′ to 3′ sens	Reverse primer written 5′ to 3′ antisens	UPL probes
*Rat Amh*	GGAGAGACTGGGGAACAGC	CAAGAGCTGAGGCTCCCATA	41
*Rat Amhr2*	CAACATCCCTTCCTCTTGGAG	CGTCCCAGCAATCTTCCA	53
*Rat Hprt*	GGTCCATTCCTATGACTGTAGATTTT	CAATCAAGACGTTCTTTCCAGTT	22
*Mouse Amh*	TCAACCAAGCAGAGAAGGTG	AGTCATCCGCGTGAAACAG	21
*Mouse Amhr2*	CCAACATCCCATCCACTTG	CTGCGTCCCAGCAATCTT	53
*Mouse Hprt*	TCAACGGGGGACATAAAAGT	CCAGTGTCAATTATATCTTCAACAATC	22
*Lhb*	ATCACCTTCACCACCAGCAT	GACCCCCACAGTCAGAGCTA	–
*Cga*	GCTGTCATTCTGGTCATGCT	GAAGCAACAGCCCATACACT	–
*Mouse Fshb*	GACAGCTGACTGCACAGGAC	CAATCTTACGGTCTCGTATACC	–
*Rat Fshb*	TTGCATCCTACTCTGGTGCT	AGCTGGGTCCTTATACAC CA	–
*Mouse Gnrhr*	CAGCTTTCATGATGGTGGTG	GGTCACACATTGCGAGAAGA	–
*Cyclophilin*	CAAAGTTCCAAAGACAGCAG	CTGGCACATGAATCCTGGAA	–

For gonadotropin subunits, *Cyclophilin* is used as reference gene for real-time PCR. Unspecified species mean that the primers are functional for both rat and mouse. For *Amh* and *Amhr2* mRNA, real-time PCR using the TaqMan PCR method was used (*Hprt* as reference gene). UPL: Universal Probe Library.
